# *Aconitum* Alkaloid Poisoning Related to the Culinary Uses of Aconite Roots

**DOI:** 10.3390/toxins6092605

**Published:** 2014-09-02

**Authors:** Thomas Y. K. Chan

**Affiliations:** Division of Clinical Pharmacology and Drug and Poisons Information Bureau, Department of Medicine and Therapeutics, Faculty of Medicine, the Chinese University of Hong Kong, Prince of Wales Hospital, Shatin, New Territories, Hong Kong, China; E-Mail: tykchan@cuhk.edu.hk; Tel.: +852-2632-3907; Fax: +852-2646-8756

**Keywords:** aconite poisoning, aconite roots, *Aconitum* alkaloids, food supplements

## Abstract

Aconite roots (roots or root tubers of the *Aconitum* species) are eaten as root vegetables and used to prepare herbal soups and meals, mainly for their purported health benefits. Aconite roots contain aconitine and other *Aconitum* alkaloids, which are well known cardiotoxins and neurotoxins. To better understand why *Aconitum* alkaloid poisoning related to the culinary uses of aconite roots can occur and characterize the risks posed by these “food supplements”, relevant published reports were reviewed. From 1995 to 2013, there were eight reports of aconite poisoning after consumption of these herbal soups and meals, including two reports of large clusters of cases (*n* = 19–45) and two reports of cases (*n* = 15–156) managed by two hospitals over a period of 4.5 to 5 years. The herbal formulae used did not adhere to the suggested guidelines, with regarding to the doses (50–500 g instead of 3–30 g per person) and types (raw instead of processed) of aconite roots used. The quantities of *Aconitum* alkaloids involved were huge, taking into consideration the doses of aconite roots used to prepare herbal soups/meals and the amounts of aconite roots and herbal soups/meals consumed. In a large cluster of cases, despite simmering raw “caowu” (the root tuber of *A. kusnezoffii*) in pork broth for 24 h, all 19 family members who consumed this soup and boiled “caowu” developed poisoning. Severe or even fatal aconite poisoning can occur after consumption of herbal soups and foods prepared from aconite roots. Even prolonged boiling may not be protective if raw preparations and large quantities of aconite roots are used. The public should be warned of the risk of severe poisoning related to the culinary and traditional medicinal uses of aconite roots.

## 1. Introduction

In Asian communities, aconite roots (roots or root tubers of the *Aconitum* species) are eaten as root vegetables and used to prepare herbal soups and meals [1,2], mainly for their purported health benefits. Unlike herbal decoctions (a popular way to administer medicinal plants), herbal soups and meals are much more palatable. Like aconite tincture [3], raw aconite plants (especially aconite roots) are highly toxic because of the presence of *Aconitum* alkaloids in very high concentrations [4,5]. Aconite roots, even after processing to markedly reduce the *Aconitum* alkaloid content, should only be used under the supervision of qualified herbalists, paying particular attention to the dosages and decoction preparation [6]. These precautionary measures are essential since *Aconitum* alkaloids are well known cardiotoxins and neurotoxins and incorrect use of processed aconite roots can result in severe or even fatal aconite poisoning [5,6,7]. It is less well known that *Aconitum* alkaloid poisoning related to the culinary uses of aconite roots can also occur.

To better understand why severe toxicity can occur after consumption of herbal soups and meals prepared from aconite roots and characterize the risks posed by these “food supplements”, relevant published reports were reviewed.

## 2. Methods

To identify relevant reports of aconite poisoning in indexed journals and Chinese medical journals, a search of Medline (1963–June 2014) and China Journal Net (1994–June 2014) was conducted, using aconite, aconitine, *Aconitum* and these terms in Chinese as the keywords. Additional articles were identified from the herbal medicine database of the Drug and Poisons Information Bureau [8]. Since the present review focused on herbal soups and meals, cases of intentional overdose, homicide and ingestion of aconite tincture, raw aconite roots and leaves and decoction of processed aconite roots were excluded. Case studies of all causes of aconite poisoning lacking separate details for subjects consuming herbal soups and meals were also excluded.

## 3. *Aconitum* Alkaloid Poisoning Related to the Culinary Uses of Aconite Roots

From 1995 to 2013, there were eight reports of *Aconitum* alkaloid poisoning after consumption of herbal soups and meals prepared from aconite roots [9,10,11,12,13,14,15,16]. There were four reports of isolated cases [9,10,11,12], two reports of large cluster of cases [13,14], and two reports of cases managed by two city hospitals in the Yunnan Province, China over a period of 4.5–5 years [15,16]. In total, six reports were from the Yunnan Province [9,12,13,14,15,16], involving 237 subjects. One report was from the Gansu Province [10] and Chongqing [11], each involving one subject. The number of subjects in each report, aconite roots involved and the presenting features are summarized in Table 1.

“Caowu” (the root tuber of *A. kusnezoffii*) [9,12,13,14,15], “chuanwu” (the root tuber of *A. carmichaeli*) [10,11] and “fuzi” (the lateral root tuber of *A. carmichaeli*) [16] were used to prepare herbal soups and meals. In four reports [9,10,11,13], raw aconite roots were used. It was not stated in other reports [12,14,15,16] whether raw or processed aconite roots were used.

**Table 1:** Reports of *Aconitum* alkaloid poisoning after consumption of herbal meals and soups.

Report	Details	
[9]	A 65-year-old man with low back pain boiled raw “caowu” 300 g and pork 1 kg in water for 4 h. He drank 200 mL of the soup and ate a small amount of pork. Twenty min later, he developed neurological symptoms, sweating, dizziness, nausea and vomiting. On arrival in hospital, he was drowsy with shock (BP 56/33 mmHg), bradycardia (50 beats/min), cyanosis and mild pulmonary edema. ECG showed frequent VEs. After resuscitation for 3 h, his condition improved. He completely recovered after a hospital stay of 3 days.	
[10] ^a^	A 57-year-old woman ingested “chuanwu”-chicken soup and 1 piece of boiled “chuanwu” (15 g). The soup was made from raw “chuanwu” and chicken using a steamer pot. Ten minutes later, she developed neurological symptoms, repeated vomiting, chest tightness and palpitations. On arrival in hospital 2 h later, she was in shock (BP 60/45 mmHg). ECG showed VEs and paroxymal VT. She was resuscitated. She was well 24 h after admission.	
[11] ^a,b^	A 50-year-old woman ingested “chuanwu”-pig leg soup (500 mL) and 1 piece of “chuanwu” (20 g). The soup was made from raw “chuanwu” and pig leg using a steamer pot. Two hours later, she developed neurological symptoms, dizziness, weakness and nausea. About 2.5 h after ingestion, she developed sweating, palpitations, cold extremities, convulsions and double incontinence. On arrival in hospital, she was in shock (BP 60/40 mmHg). ECG showed frequent AEs. She had multiple episodes of VT and 1 episode of bradycardia. She completely recovered.	
[12] ^a,c,d^	A 39-year-old woman and her husband drank 200–250 mL and 400–500 mL of “caowu”-pig leg soup at home. Her husband woke up from sleep with neurological symptoms. She was then found to be unarousable. Despite resuscitation in hospital, she died 6 h after ingestion. Aconitine was found to be present in gastric tissues, gastric contents and the liver.	
[13]	All 19 family members who had ingested “caowu” soup at dinner in a hotel developed mild (*n* = 12) or moderate to severe (*n* = 7) symptoms. The soup was made by simmering raw “caowu” (3–7 cm long, 0.5–2.0 cm in diameter, 20–30 g each) in pork broth for 24 h and reheated before consumption. Seven subjects (aged 32–80 years) ingested 4–13 pieces of boiled “caowu” and 50–200 mL of soup. They developed mild neurological symptoms, blurred vision and palpitations, while 4 subjects also reported nausea and vomiting. Symptoms appeared 10–40 min (mean 30 min) after ingestion. Six subjects completely recovered after in-hospital treatment. An 80-year-old woman who ingested 13 pieces of boiled “caowu” and 200 mL of soup died in hospital. Twelve subjects who ingested <3 pieces of boiled “caowu” and a smaller amount of soup had mild neurological symptoms not requiring treatment.	
[14] ^a,c,e^	Thirty-two men and 13 women, aged 30–57 years, developed aconite poisoning within 1–5 h of eating pork ribs cooked with “caowu” in a restaurant. Their main complaints were neurological symptoms (*n* = 45), nausea/vomiting (*n* = 43), chest discomfort/palpitations (*n* = 21), abdominal pain (*n* = 19), fall in BP (*n* = 15), supraventricular tachycardia (*n* = 8), VEs (*n* = 7), sinus bradycardia (*n* = 5), shock (*n* = 3) and right bundle branch block (*n* = 2). The dose of “caowu” ingested was from <5 g (*n* = 10), 5–10 g (*n* = 26) to >10 g (*n* = 9). All 45 subjects fully recovered after a hospital stay of 1–8 days.	
[15] ^a,c,e^	During 2004–2008, 9 men and 6 women, aged 28–72 years, presented to hospital with aconite poisoning after eating “caowu” cooked in pork broth. They arrived in hospital 0.5–3 h after the onset of symptoms. The main clinical features included convulsions (*n* = 6), irritability (*n* = 4) and VT plus severe gastrointestinal symptoms (*n* = 2). Three patients died from severe cardiac arrhythmias, central nervous system and respiratory depression and circulatory failure.
[16] ^a,c,e,f^	During 2006–2010, 123 men and 33 women, aged 25–68 years, were admitted to hospital with aconite poisoning after ingesting herbal soups or meals containing “fuzi” 50–500 g. Symptoms appeared within 0.5–2.0 h. These included neurological symptoms, irritability, weakness, slurred speech, dizziness, nausea, vomiting, abdominal pain, palpitations, shock, chest tightness and difficulty in breathing. ECG showed ventricular arrhythmias (*n* = 142) and supraventricular tachycardia (*n* = 41). Sinus bradycardia and first-, second- and third-degree atrioventricular block. In 155 subjects, cardiac arrhythmias subsided after 2–16 h of resuscitation; they stayed in hospital for 2–4 days. One subject died of refractory cardiac arrhythmias.

“Caowu” (the root tuber of *A. kusnezoffii*); “chuanwu” (the root tuber of *A. carmichaeli*); “fuzi” (the lateral root tuber of *A. carmichaeli*); BP = blood pressure; VE = ventricular ectopics; VT = ventricular tachycardia; AE = atrial ectopics; neurological symptoms = paresthesia/numbness of the face, perioral area and the four limbs; ^a^ The duration of boiling or cooking of aconite roots was not stated; ^b^ Presumably, boiled aconite root was ingested; ^c^ It was not stated if raw or processed aconite roots had been used for cooking; ^d^ It was not stated if boiled aconite roots were also ingested; ^e^ The amounts of herbal soups, boiled aconite roots and meat ingested per person were not stated; ^f^ The number of subjects with VE, VT and ventricular fibrillation was not stated.

In two reports [9,16], the doses of aconite roots used to prepare herbal soups and meals were mentioned (50–500 g per person). In two large clusters of cases [13,14], the amounts of aconite roots used were much larger, taking into consideration the size of the soups and meals for 19–45 people and the amounts of boiled “caowu” ingested per person.

In two reports [9,13], the method and duration of cooking of herbal soups and meals were mentioned, ranging from 4 h in an isolated case to 24 h in a large cluster of cases.

In six reports, mainly herbal soups [9,12] and herbal soups with boiled aconite roots [10,11,13,15] were ingested. In one report, mainly herbal meal (pork ribs) was ingested [14]. In one report, the relative quantities of herbal soups and meals consumed were not mentioned [16]. In two large clusters of cases [13,14], subjects requiring in-hospital treatment each ingested 1–13 pieces (each weighing 20–30 g) or <5 to >10 g of boiled “caowu”, in addition to the herbal soups or meals.

In four reports of isolated cases [9,10,11,12], aconite poisoning was either severe (with life-threatening complications including shock and ventricular tachycardia) or fatal (Table 1). In a cluster of 19 cases from one family [13], 1 of the 7 subjects requiring in-hospital treatment died. In a cluster of 45 cases [14], 3 subjects were in shock. In two reports of cases managed by two city hospitals [15,16], the mortality rate was 0.6%–20%.

## 4. Discussion

In Asian communities, herbal soups and meals are popular, especially in late autumn and early winter. Many people believe that foods are good alternatives to medicines as health tonics and medicinal plants and foods should be eaten together to maximize their beneficial effects on health [17]. The popularity of herbal soups and meals is reflected by the listing of >7300 formulae in the “Chinese Medicated Diet Dictionary” and their inclusion in the menu of some restaurants [13,14,18].

Aconite roots are used to prepare herbal soups and meals for their purported beneficial effects on human health, e.g., to dispel “wind”, remove “dampness”, relieve pain, improve immunity and physical strength and to serve as health tonics [15]. Herbal soup prepared from processed “fuzi” 4 g, chicken and other herbs by simmering for two hours is a suggested treatment for cold hands and feet [19]. Lamb cooked with processed “fuzi” 6 g and spices is a suggested treatment for bowel dysfunction [20]. The “Chinese Medicated Diet Dictionary” lists eight other recipes of herbal soups and meals prepared from processed “fuzi” 3–30 g [18].

As can be seen in Table 1, the herbal formulae responsible for aconite poisoning did not adhere to the suggested guidelines [18,19,20], with regarding to the doses (50–500 g instead of 3–30 g per person) [9,16] and types (raw instead of processed) of aconite roots used [9,10,11,13]. The amounts of *Aconitum* alkaloids involved were huge, taking into consideration the doses of aconite roots used to prepare these herbal soups and meals [9,13,14,16] and the quantities consumed per person [9,10,11,14,16]. Prolonged boiling should markedly reduce the toxicity of both aconite roots and herbal soups through hydrolysis of *Aconitum* alkaloids to less toxic and non-toxic derivatives [5,6,7]. However, despite simmering raw “caowu” in pork broth for 24 h, all 19 family members who ingested this soup and boiled “caowu” developed aconite poisoning and the severity of symptoms was related to the quantities consumed [13]. This poisoning outbreak well illustrated the toxic potential of herbal soups and meals prepared from aconite roots. Even prolonged boiling might not be protective if raw preparations and large quantities of aconite roots were used.

**Table 2:** Differences in practice between the culinary and traditional medicinal uses of aconite roots.

Differences in practice	“Food supplements” (herbal soups/meals)	Traditional medicines (herbal decoction)
Types of aconite roots		
Recommended	Processed ^a^	Processed ^b^
Actual use	Raw ^c^	Processed ^b^
Doses of aconite roots		
Recommended	“Caowu”—not available	“Caowu” 1.5–3 g ^b^
	“Chuanwu”—not available	“Chuanwu” 1.5–3 g ^b^
	“Fuzi” 3–30 g ^a^	“Fuzi” 3–15 g ^b^
Actual use	“Caowu” <5–390 g ^d^	“Caowu” 7–30 g ^b,e^
	“Chuanwu”—not available	“Chuanwu” 7–30 g ^b,e^
	“Fuzi” 50–500 g ^f^	“Fuzi” 6 g ^e,g^
Supervision by herbalists	No	Strongly recommended ^h^

^a^ [18,19,20]; ^b^ [5,7]; ^c^ [9,10,11,13]; ^d^ [9,13,14]; ^e^ In non-fatal cases [5] the doses involved were 7–11 g per aconite root and 14–22 g per prescription. In fatal cases [7], the doses involved were 6–30 g per aconite root and 9–60 g per prescription. Limited data about “fuzi” was available from a systematic review of published reports of herb-induced aconite poisoning [7]; ^f^ [16]; ^g^ [7]; ^h^ Unsupervised use of aconite roots might often result in overdose and improper decoction preparation and hence aconite poisoning [5,6,7].

The cardiotoxicity and neurotoxicity of *Aconitum* alkaloids can be explained by their actions on the voltage-sensitive sodium channels of the cell membranes of excitable tissues, including myocardium, nerves and muscles [5,6,7]. In addition, aconitine has been shown to inhibit the amplitude of voltage-dependent potassium current in a time-, concentration- and state-dependent manner [21,22]. Thus, it is hypothesized that both actions of aconitine may act synergistically to affect the functional activity of the excitable tissues [21,22]. Patients with aconite poisoning generally present with a combination of neurological, cardiovascular, gastrointestinal, and other features (Table 1). The main causes of death are refractory ventricular tachyarrhythmias and asystole [5,6,7].

There are major differences in practice between the culinary (“food supplements”) and traditional medicinal uses of aconite roots when they are used respectively to prepare herbal soups/meals and decoction (Table 2). In general, herbal soups and meals are associated with a higher risk of severe or fatal poisoning because of the use of raw preparations and large doses of aconite roots. In the absence of supervision from the herbalists, incorrect use and overdose of aconite roots are particularly likely to occur [5,6].

## 5. Conclusions

Aconite roots are eaten as root vegetables and used to prepare herbal soups and meals, mainly for their purported beneficial effects. Severe or even fatal *Aconitum* alkaloid poisoning can occur after consumption of herbal soups and foods prepared from aconite roots. Even prolonged boiling may not be protective if raw preparations and large quantities of aconite roots are used. The public should be warned of the risk of severe poisoning related to the culinary and traditional medicinal uses of aconite roots.
